# Exploring GPs’ assessments of their patients’ cancer diagnostic processes: a questionnaire study

**DOI:** 10.3399/BJGP.2022.0651

**Published:** 2023-10-31

**Authors:** Gitte Bruun Lauridsen, Dorte Ejg Jarbøl, Peter Thye-Rønn, Sanne Rasmussen, Kirubakaran Balasubramaniam, Jesper Lykkegaard

**Affiliations:** Research Unit of General Practice, Department of Public Health, University of Southern Denmark, Odense.; Research Unit of General Practice, Department of Public Health, University of Southern Denmark, Odense.; Department of Clinical Research, Faculty of Health Sciences, University of Southern Denmark, Odense, and Diagnostic Center, Svendborg Hospital, Svendborg.; Research Unit of General Practice, Department of Public Health, University of Southern Denmark, Odense.; Research Unit of General Practice, Department of Public Health, University of Southern Denmark, Odense.; Department of Public Health and Audit Project Odense, Research Unit of General Practice, University of Southern Denmark, Odense.

**Keywords:** early detection of cancer, general practice, signs and symptoms

## Abstract

**Background:**

Most cancer diagnostic pathways start from primary care and several factors affect the diagnostic processes.

**Aim:**

To analyse the associations between patient characteristics, symptom presentation, and cancer type and the GP’s assessment of the diagnostic processes.

**Design and setting:**

General practices in the North, Central, and Southern regions of Denmark were invited to participate in a questionnaire survey.

**Method:**

Participating GPs received a list of patients with incident cases of cancer in the period between 1 March 2019 and 28 February 2021 based on administrative hospital data. A questionnaire was completed for each patient, addressing symptom presentation and the GP’s assessment of the diagnostic process both overall and in four subcategories (the patient’s role, the GP’s role, the transition between primary and secondary care, and the secondary sector’s role).

**Results:**

A total of 187 general practices informed on 8240 patients. For 5868 patients, diagnostic pathways started in general practice. Almost half (48.3%, 2837/5868) presented with specific cancer symptoms. GPs assessed 55.6% (3263) and 32.3% (1897) of the diagnostic processes as ‘very good’ and ‘predominantly good’, respectively; 11.9% (700) were ‘predominantly poor’ or ‘very poor’ for these 5868 patients. Long symptom duration of ≥2 months prior to GP contact and presenting with non-specific or a combination of non-specific and specific symptoms were associated with a poor overall assessment of the diagnostic process. Assessment in the four subcategories showed that the patient’s role was assessed less positively than the other three categories.

**Conclusion:**

A longer symptom duration and presenting without cancer-specific symptoms were associated with GPs assessing the diagnostic process as poor.

## INTRODUCTION

Cancer diagnostic pathways vary internationally, affecting timeliness of diagnosis.^1^^,^^2^ As timely diagnosis can improve treatment options and reduce mortality, it is important to investigate factors affecting these pathways.^3^^,^^4^ Delays in the pathway from first symptom to cancer treatment can be divided into those relating to the patient, the GP, and the system interval.^5^^,^^6^

A prerequisite for timely cancer diagnosis is that the patients seek medical attention for their symptoms. A UK study found that 72% of patients with a new cancer diagnosis had their first contact in general practice, and 94% had at least one recorded symptom.^7^ Not all symptoms indicative of cancer lead to GP contact^8^^,^^9^ and several factors can influence healthcare-seeking.^10^

Another prerequisite is the initiation of investigations by the GP when symptoms indicate possible cancer. Diagnosing cancer is complex because it is a relatively rare diagnosis in general practice and patients may present with a range of symptoms that may also be caused by benign conditions.^11^

In Denmark, most healthcare services are tax paid. Nearly all citizens are listed with a general practice and the GPs act as gatekeeper to other healthcare providers, including the cancer diagnostic pathways.^2^ Patients presenting with specific cancer symptoms should be referred to a specific ‘cancer patient pathway’ (CCP).^4^^,^^12^ However, no more than half of patients with cancer present with these. The remaining present with non-specific or no symptoms and can be referred for a *‘non-specific signs and symptoms of cancer patient pathway’* (NSSC-CCP).^2^^,^^13^^–^^15^ The cancer diagnostic process is diverse despite national guidelines, as it is based on the GP’s subjective evaluation of the patients. Moreover, it is influenced by several factors and is often not linear.^16^^,^^17^

In this study, the diagnostic pathway from symptom to diagnosis is examined by the GP both overall and in subcategories evaluating the role of the patient, the GP, and the secondary sector, as well as the sector transition from primary to secondary care. The GP assessment is subjective and can be based on a number of parameters such as events experienced, number of consultations before referral, time to diagnosis, and whether the process, in general, is found to be acceptable, several of which are important to achieve a timely diagnosis. The GP assessment of the diagnostic pathway can therefore possibly be presumed a proxy for timely diagnosis.

**Table:** How this fit in

The cancer diagnostic process has improved considerably over past years as a response to both the introduction of a pathway for patients with cancer and increased diagnostic workup. Still, more than one in 10 of cancer diagnostic processes are overall assessed as poor by the GP. Long symptom duration before healthcare-seeking and presenting with non-specific symptoms remains a large challenge. Better ways of managing these patients in general practice are desirable.

The aim of this study was to explore the association between demographics, symptom presentation and duration, cancer type and the GP’s assessment of the diagnostic processes, overall and in four subcategories for patients with cancer.

## METHOD

### Design

In total, 852 general practices in the Regions of Southern, Central, and North Denmark received postal invitations to this questionnaire survey, based on the Audit Project Odense (APO) method.^18^ The APO method implies that the participating GPs register case details, their thoughts, and actions each time a recurrent issue occurs and subsequently meet to discuss the results.^18^

### Study population

All patients with incident cases of cancer (except non-melanoma skin cancers) in the period between 1 March 2019 and 28 February 2021 who were enlisted with a participating practice were eligible for the study. Data were extracted from each region’s patient records using International Classification of Diseases, 10th Revision cancer codes. Most follow-up programmes after cancer will terminate after 5 years without recurrence. Therefore, patients were considered newly diagnosed if they had no similar diagnosis in the preceding 5 years.

**Figure 1.**
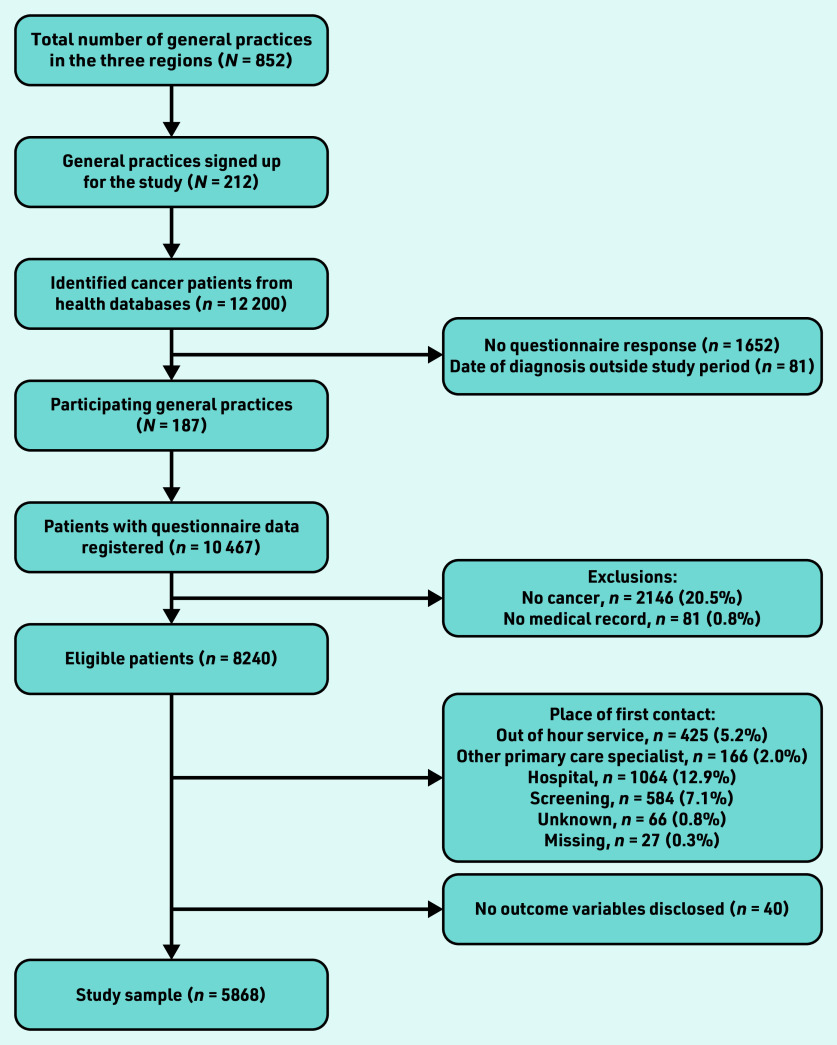
*Flow of participants.*

GPs verified the cancer diagnoses based on their medical records. Only patients with cancer with a first contact in general practice were included (Figure 1).

### Data collection

A complete list of all patients with cancer identified in the given practice, including consecutive serial number, name, personal identification number, cancer type, and date of diagnosis, was sent from the regional government authority to each practice via secure electronic mail. At the end of the study period GPs were asked to complete a questionnaire for each patient. Instructions and a list of symptoms were distributed to the GPs prior to the study (symptom list in Supplementary Table S1).

### Questionnaire development

The questionnaire was designed to investigate the following constructs: place of first contact with signs or symptoms that could be because of cancer, date of first contact in general practice, symptom duration before GP contact, symptom presentation, and assessment of the overall diagnostic process and the four subcategories (the patient’s, the GP’s, and the secondary sector’s role, and the transition between primary and secondary care).

Development followed these steps:^19^

- a literature search exploring the concepts of cancer diagnostic processes;^2^^,^^4^^,^^12^^,^^20^
- interviews with GPs and heads of the cancer diagnostic centres in the Region of Southern Denmark;
- conceptualisation and operationalisation by the project group;
- a qualitative pilot test with observed responses; and
- a pilot test where two GPs tested time consumption, content validity, acceptability, and feasibility, along with a quality assessment of the data extraction.

In the current study, symptom duration was defined as the time from the first symptom experience to first presentation (patient interval).^5^ Symptom presentation was reported as either non-specific, cancer specific, or no symptoms.^21^^,^^22^ ‘No symptoms’ covers patients without symptoms related to cancer that may have consulted the GP for another reason, for example, a yearly control of chronic illness, or another health issue, where the GP incidentally became aware of a sign or an abnormal test result. A codebook of all data and their coding is provided in Supplementary Table S2.

### Analyses

The primary outcome variable is the GP’s assessment of the diagnostic process on a four-point Likert scale: 1, ‘very good’; 2, ‘predominantly good’; 3, ‘predominantly poor’; and 4, ‘very poor’. For analyses, responses were dichotomised into: 1, overall good (very good/predominantly good) and 2, overall poor (very poor/predominantly poor).

Descriptive variables are: patient characteristics (age and sex); cancer type; symptom duration (in weeks); symptom presentation in the first consultation; and region. Both age groups and symptom duration were categorised with arbitrary cut-offs.

Descriptive data are presented as numbers and percentages. Associations between the independent variables and the GP’s assessment of the overall process and the four subcategories were tested for statistical significance with robust cluster regression analyses adjusted for age, sex, cancer type, region, and symptom presentation, reported as odds ratios (ORs) with 95% confidence intervals (CIs). An interaction analysis was carried out exploring interaction effects between symptom duration and cancer type on the assessment of the diagnostic process, finding no significant interactions overall. Moreover, analysis of each region was conducted to check for comparability. All analysis was conducted in Stata Statistical Software Release 17.

## RESULTS

A total of 187 (21.9%) of the 852 general practices, listed in the three regions, participated. Some 12 119 patients with a new cancer diagnosis from March 2019 to April 2021 were registered in the corresponding hospitals’ administrative records. Completed questionnaire data were received for 10 467 patients. Exclusion of patients with no new cancer diagnosis, no available medical record according to the GPs, and patients for whom general practice was not the first place of contact or without a record of GP assessment disclosed resulted in a total study sample of 5868 patients (Figure 1).

Cancer frequency was slightly higher in males (52.5%, 3081/5868). Most of the patients were 61–80 years old (59.1% 3468/5868). A total of 8.8% (517/5868) of the patients contacted the GP within 0–6 days and additionally, 24.3% (1426/5868) within 1–3 weeks. Most patients presented with a specific cancer symptom (48.3%, 2837/5868). A total of 12.8 (752/5868) of patients presented with no symptoms related to the cancer (Table 1).

**Table 1.** Characteristics of the study sample (*n* = 5868)

**Characteristic**	**Number (%)**
**All**	5868 (100)

**Sex**	
Female	2787 (47.5)
Male	3081 (52.5)

**Age groups, years**	
0–40	294 (5.0)
41–60	1145 (19.5)
61–80	3468 (59.1)
>80	961 (16.3)

**Symptom duration**	
0–6 days	517 (8.8)
1–3 weeks	1426 (24.3)
4–8 weeks	973 (16.6)
2–6 months	513 (8.7)
>6 months	347 (5.9)
Not disclosed^a^	2092 (35.7)

**Symptom presentation**	
No symptoms presented	752 (12.8)
Only specific cancer symptoms	2837 (48.3)
Only non-specific symptoms	2006 (34.2)
Both specific cancer and non-specific symptoms	273 (4.7)

**Cancer type (by group)**	
Head and neck cancer	167 (2.8)
Lung cancer	780 (13.3)
Breast cancer	737 (12.6)
Upper gastrointestinal cancer	219 (3.7)
Pancreas, liver, and gallbladder cancer	325 (5.5)
Lower gastrointestinal cancer	671 (11.4)
Urological cancer	328 (5.6)
Gynaecological cancer	285 (4.9)
Male genitalia/reproductive organs	971 (16.5)
Haematological cancer	511 (8.7)
Malignant melanoma	544 (9.3)
Other	330 (5.6)

**Region**	
Region of Southern Denmark	2065 (35.2)
Region of Central Denmark	3032 (51.7)
Region of North Denmark	771 (13.1)

a *Not disclosed if the question was left blank on the questionnaire or marked as ‘unknown’.*

Most patients presented with a specific cancer symptom (48.3%, 2837/5868). A total of 12.8% (752/5868) of patients presented with no symptoms related to the cancer (Table 1). GPs assessed 55.6% (3263/5868) and 32.3% (1897/5868) of the diagnostic processes as ‘very good’ and ‘predominantly good’, respectively; 11.9% (700/5868) were ‘predominantly poor’ or ‘very poor’.

Assessment in the four subcategories showed that the GP’s role, the transition between sectors, and the secondary sector’s role were assessed more positively than the patient’s role (Table 2). Descriptive data are shown in Supplementary Table S3.

**Table 2.** GPs’ assessment of the diagnostic process (*n* = 5868)

**Evaluation**	**Overall assessment, *n* (%)**	**Assessment of the patient’s role, *n* (%)**	**Assessment of the GP’s role, *n* (%)**	**Assessment of the sector transition, *n* (%)**	**Assessment of the secondary sector’s role, *n* (%)**
**Very good**	3263 (55.6)	3620 (61.7)	3977 (67.8)	4487 (76.5)	4359 (74.3)
**Predominantly good**	1897 (32.3)	1493 (25.4)	1515 (25.8)	1026 (17.5)	1065 (18.1)
**Predominantly poor**	579 (9.9)	546 (9.3)	324 (5.5)	250 (4.3)	286 (4.9)
**Very poor**	121 (2.1)	186 (3.2)	45 (0.8)	90 (1.5)	103 (1.8)
**Missing**	8 (0.1)	23 (0.4)	7 (0.1)	15 (0.3)	55 (0.9)

The adjusted analyses of associations between patient characteristics, symptom presentation and duration, cancer type, region, and the assessments of the diagnostic process are shown in Table 3 (with crude analyses in Supplementary Table S4).

**Table 3.** Associations between sex, age, symptom presentation, symptom duration, and cancer type and assessment of the diagnostic process, shown by overall assessment, and assessment of the patient’s role, the GP’s role, the sector transition, and the secondary sector’s role^a^

**Characteristic**	**Adjusted OR (95% CI)**				

	**Overall assessment (*n* = 5860)**	**Assessment of the patient’s role (*n* = 5845)^b^**	**Assessment of the GP’s role (*n* = 5861)^b^**	**Assessment of the sector transition (*n* = 5853)^b^**	**Assessment of the secondary sector’s role (*n* = 5813)^b^**
**Sex**					
Female	Reference	Reference	Reference	Reference	Reference
Male	1.08 (0.91 to 1.29)	**0.69 (0.57 to 0.83)**	1.03 (0.79 to 1.34)	1.16 (0.91 to 1.47)	1.11 (0.88 to 1.41)

**Age groups (years of age)**					
0–40	0.90 (0.57 to 1.43)	1.18 (0.71 to 1.96)	0.89 (0.48 to 1.65)	1.12 (0.59 to 2.13)	0.95 (0.57 to 1.59)
41–60	Reference	Reference	Reference	Reference	Reference
61–80	0.98 (0.78 to 1.23)	0.86 (0.66 to 1.11)	0.92 (0.67 to 1.25)	0.96 (0.69 to 1.33)	1.17 (0.90 to 1.52)
> 80	0.88 (0.67 to 1.16)	**0.73 (0.57 to 0.94)**	0.71 (0.51 to 1.00)	1.02 (0.70 to 1.48)	1.23 (0.86 to 1.76)

**Symptom duration**					
0–6 days	Reference	Reference	Reference	Reference	Reference
1–3 weeks	1.04 (0.72 to 1.49)	1.32 (0.80 to 2.18)	0.72 (0.44 to 1.18)	1.13 (0.71 to 1.79)	1.44 (1.00 to 2.08)
4–8 weeks	0.73 (0.51 to 1.06)	**0.54 (0.36 to 0.81)**	0.69 (0.42 to 1.12)	1.02 (0.67 to 1.55)	1.12 (0.75 to 1.68)
2–6 months	**0.58 (0.36 to 0.93)**	**0.18 (0.11 to 0.30)**	0.55 (0.29 to 1.03)	1.24 (0.66 to 2.33)	1.41 (0.82 to 2.44)
>6 months	**0.34 (0.21 to 0.53)**	**0.11 (0.07 to 0.18)**	**0.43 (0.23 to 0.81)**	0.76 (0.46 to 1.24)	0.79 (0.47 to 1.33)
Not disclosed	**0.70 (0.49 to 0.99)**	**0.51 (0.33 to 0.78)**	**0.59 (0.35 to 0.97)**	1.21 (0.78 to 1.86)	1.38 (0.94 to 2.04)

**Symptom presentation**					
None	0.94 (0.67 to 1.31)	1.17 (0.88 to 1.56)	0.73 (0.51 to 1.06)	0.73 (0.52 to 1.03)	1.01 (0.67 to 1.51)
Only specific cancer symptoms	Reference	Reference	Reference	Reference	Reference
Only non-specific symptoms	**0.61 (0.50 to 0.74)**	0.88 (0.73 to 1.07)	**0.56 (0.42 to 0.75)**	**0.57 (0.44 to 0.75)**	**0.63 (0.48 to 0.81)**
Both specific cancer and non-specific symptoms	**0.53 (0.37 to 0.74)**	**0.55 (0.37 to 0.83)**	**0.52 (0.31 to 0.88)**	**0.48 (0.29 to 0.80)**	**0.48 (0.31 to 0.74)**

**Cancer type (by group)**					
Head and neck cancer	**0.44 (0.25 to 0.79)**	**0.58 (0.35 to 0.96)**	**0.37 (0.15 to 0.88)**	**0.39 (0.18 to 0.87)**	**0.49 (0.25 to 0.97)**
Lung cancer	**0.43 (0.29 to 0.64)**	0.78 (0.55 to 1.10)	**0.49 (0.25 to 0.93)**	0.61 (0.32 to 1.15)	**0.57 (0.33 to 0.98)**
Breast cancer	Reference	Reference	Reference	Reference	Reference
Upper gastrointestinal cancer	**0.29 (0.18 to 0.49)**	0.83 (0.51 to 1.34)	**0.39 (0.17 to 0.89)**	**0.36 (0.18 to 0.71)**	**0.49 (0.27 to 0.90)**
Pancreas to liver and gallbladder cancer	**0.28 (0.18 to 0.45)**	1.09 (0.69 to 1.73)	**0.33 (0.15 to 0.72)**	**0.33 (0.17 to 0.62)**	**0.30 (0.18 to 0.52)**
Lower gastrointestinal cancer	**0.46 (0.31 to 0.68)**	0.77 (0.56 to 1.07)	**0.45 (0.24 to 0.81)**	0.64 (0.38 to 1.08)	0.65 (0.39 to 1.09)
Urological cancer	**0.37 (0.23 to 0.61)**	0.91 (0.59 to 1.40)	**0.41 (0.19 to 0.89)**	**0.31 (0.15 to 0.62)**	**0.38 (0.20 to 0.73)**
Gynaecological cancer	0.73 (0.43 to 1.23)	0.93 (0.58 to 1.47)	0.77 (0.33 to 1.82)	0.78 (0.36 to 1.70)	0.70 (0.36 to 1.35)
Male genitalia/reproductive organs	0.90 (0.56 to 1.44)	**2.87 (1.83 to 4.50)**	0.69 (0.34 to 1.42)	0.71 (0.37 to 1.37)	0.74 (0.41 to 1.34)
Haematological cancer	**0.57 (0.36 to 0.89)**	**1.63 (1.06 to 2.52)**	**0.42 (0.22 to 0.80)**	**0.44 (0.25 to 0.80)**	**0.55 (0.34 to 0.90)**
Malignant melanoma	0.72 (0.44 to 1.18)	1.27 (0.88 to 1.84)	0.54 (0.28 to 1.03)	0.70 (0.38 to 1.26)	1.11 (0.63 to 1.96)
Other	**0.45 (0.28 to 0.71)**	0.77 (0.50 to 1.19)	**0.39 (0.18 to 0.84)**	**0.36 (0.20 to 0.63)**	**0.47 (0.28 to 0.78)**

**Region**					
Region of Southern Denmark	Reference	Reference	Reference	Reference	Reference
Region of Central Denmark	1.10 (0.82 to 1.49)	1.13 (0.83 to 1.54)	0.97 (0.66 to 1.43)	0.88 (0.57 to 1.37)	1.01 (0.68 to 1.51)
Region of North Denmark	0.83 (0.59 to 1.17)	0.81 (0.57 to 1.14)	**0.62 (0.39 to 1.00)**	0.74 (0.42 to 1.29)	0.74 (0.45 to 1.20)

a *Robust cluster regression analyses adjusted for gender, age, region, cancer type and symptom presentation. Bold indicates significant values P<0.05.Count (n) is not 5868 because of missing values in the five assessments of the diagnostic process.*

b *(*n *= 5845), (n = 5861), (*n *= 5853), and (*n *= 5813) account for the missing outcome data shown in the table. CI = confidence interval. OR = odds ratio.*

Regarding the overall assessment, no significant associations were found with the patient’s sex and age. Symptom duration of ≥2 months and presenting with non-specific symptoms or both specific and non-specific symptoms were associated with poorer assessment of the diagnostic process.

Regarding the patient’s role, both male sex and older age were significantly associated with poorer assessments of the diagnostic process (adjusted OR [OR_adj_] 0.69, 95% CI = 0.57 to 0.83 and OR_adj_ 0.73, 95% CI = 0.57 to 0.94, respectively). Also, a symptom duration of ≥4 weeks and presenting with a combination of both specific- and non-specific symptoms were associated with significantly poorer assessments of the patient’s role. Compared with breast cancer, cancer in male genitalia and haematological cancer were associated with an increased likelihood of a good assessment of the patient’s role (OR_adj_ 2.87, 95% CI = 1.83 to 4.50 and OR_adj_ 1.63, 95% CI = 1.06 to 2.52) (Table 3).

Regarding the GP’s role, the sector transition, and the secondary sector’s role, neither age nor sex was significantly associated with assessment of the diagnostic process. Presenting with non-specific symptoms or a combination of both specific and non-specific symptoms was associated with poorer assessment of the diagnostic process in all three of these subcategories. The symptom duration was only significantly associated with poor assessment of the GP’s role. Finally, most other cancers increased the likelihood of a poor assessment when compared with breast cancer, although not statistically significant for cancer of male genitalia, gynaecological cancers, and malignant melanoma (Table 3).

In the assessment of the GP’s role, practising in the Region of Northern Denmark was associated with a poorer diagnostic process compared with Southern Denmark (OR_adj_ 0.62, 95% CI = 0.39 to 1.00) (Table 3).

For 557 patients, the GP had recorded ‘No symptoms’ but also indicated a symptom duration of ≥1 week. A sensitivity analysis excluding these patients from analyses showed no significant difference to the main analyses (data not shown).

## DISCUSSION

### Summary

Although most of the diagnostic processes were assessed to be good overall, some 12% were not. Patients presenting with non-specific symptoms had higher odds of their diagnostic course being assessed as poor. This applied to both the overall assessment and all four subcategories. Moreover, a long symptom duration was associated with poor assessment, both overall and of the patient’s and the GP’s roles in the process, whereas older age (>80) and male sex were only significantly associated with assessment of the patient’s role. Compared with breast cancer, most other cancers were associated with a poorer assessment of the different aspects of the diagnostic processes.

### Strengths and limitations

Inclusion of patients in this study via the regions’ patient records was highly complete and consistent. A rather large number of patients were subsequently excluded from the present study as the focus was on patients who first presented in primary care. Misclassification in terms of date of diagnosis or diagnostic code cannot be ruled out; this potential bias is presumably minimal, given the quality of Danish health databases and the preceding pilot testing of the data extraction. Although the study population was rather large there were 1652 patients for whom the questionnaire was not returned (Figure 1). Those patients may have had difficult or uneven diagnostic processes. The missing questionnaire data may be from individuals with difficult or uneven diagnostic processes. In which case, this study may have underestimated the number of patients with a poor diagnostic course. GPs’ participation was voluntary, which may indicate that participating GPs may have already had an increased focus on the topic.^23^ This could both mean an under- and overestimation of the number of poor diagnostic processes. The increased focus could be because of both a lack of knowledge and organisational challenges in the management of cancer. On the other hand, the GP might be more educated about the topic because of the increased focus.

The questionnaire survey was based on information held by the GP up until 2 years after the diagnosis, including medical records. Thus, recall bias cannot be eliminated, for example, it is not possible to exclude underreporting of vague symptoms because of a focus on symptoms relevant to each patient’s cancer type. However, the findings from the study reflect previous findings regarding symptom recording. Moreover, one should keep in mind that all parts of the diagnostic process were assessed by the GP. The diagnostic processes were not assessed by the patients or colleagues in the secondary sector.

The GP was asked to assess the diagnostic process on a Likert scale. There was no ‘neither good nor poor option’ as in this study the authors wanted the GP to reflect on each of the diagnostic processes. This may have caused an underestimation of ‘true poor diagnostic processes’ because of self-report bias.

In some diagnostic processes, the patient does not have any presenting symptoms, which may have challenged the GPs’ assessment of the patient’s and the GP’s role in the process. However, in these patients the assessment could, for instance, depend on how the patient and the GP acted on the incidental findings: Did the patient agree to the investigative plan? Did the GP initiate a further investigation? And so forth.

Most published cancer studies are organ specific, which is challenging for comparison purposes.^3^ In the current study, the authors investigated assessment of the diagnostic process on a broad range of cancer types, providing non-organ specific findings, allowing adjustment not only for sex, age, and presenting symptoms, but also for cancer type.

### Comparison with existing literature

From the qualitative pilot test, the authors learned that GPs based their assessments on the timeliness of and events experienced during the diagnostic process, as well as the general quality of the overall diagnostic process. A previous study supports the importance and usability of GPs’ gut feelings, describing how this especially can be useful in ‘grey areas’ of the diagnostic process.^24^

Quality deviations can occur in diagnostic pathways.^25^ Kostopoulou *et al* found that atypical or non-specific presentation, low prevalence, and comorbidity were some of the factors associated with difficulties in cancer diagnostic pathways.^26^ In 2014, Jensen *et al* found that involving patients with non-specific symptoms was more likely to involve suboptimal clinical decisions.^27^ These findings are consistent with the present study, indicating that the diagnostic challenges of non-specific cancer symptoms remain to be solved.

As with previous studies in Danish primary care, the current study found that 48.3% of patients presented with specific cancer symptoms.^13^^,^^14^ Koo *et al* found that a third of all cancer patients present with ≥2 symptoms in the UK.^3^ Moreover, presenting with non-specific symptoms was associated with increased odds of stage IV disease.^3^ This is supported by a systematic review from 2022.^28^

The current study found that older age (>80) was associated with a poorer assessment by the GP of the patient’s and the GP’s role but not the transition or secondary sector’s role. The older patients most likely had more difficult symptom presentation to express and interpret, more comorbidities, less desire for diagnostic investigations, and higher risk of most cancer types. In a register-based cohort study, older individuals were more likely to be diagnosed via an unplanned admission, death certificate only, and outpatient admission, compared with younger patients.^29^ A reason for the GPs’ assessments might be that GPs accept the professional responsibility for the more troublesome cancer diagnostic processes with older compared with younger patients.

Previous studies have found delays in different intervals of the diagnostic pathway, stating that most delays stemmed from patient and system intervals.^7^^,^^30^ In the present study, nearly one in four had a symptom duration of 1–3 weeks, whereas one in six had a duration of 4–8 weeks. For 35.7% of the patients, the symptom duration was not reported. This might be because the patient did not have any symptoms at the time of the first contact, or because the GP did not have any records regarding the symptom duration.

In the Region of Northern Denmark, the current study found a small difference in the assessment of the diagnostic process regarding the GP’s role compared with the GP assessment in the Region of Southern Denmark. Even though Denmark is a small country there are some differences in patients’ access to healthcare services as well as the GPs’ options for requesting investigations for patients with non-specific signs and symptoms of cancer.^31^

Coincidently, the study period was almost exactly 1 year before and after the first Danish COVID-19 lockdown, starting 11 March 2020. Previous studies have found that healthcare-seeking behaviour changed at the beginning of the pandemic, with lower use of general practice and an increase in remote consultations^32^^,^^33^ resulting in fewer cancer diagnoses during the COVID-19 lockdown.^34^^,^^35^ These findings are in accordance with the present study, where fewer cancer diagnoses were found in the year after the onset of COVID-19. Despite the possible challenges in cancer diagnostic processes in 2020, the effect on the assessment is probably minimal since the GP can take the entire period into account.

### Implications for research and practice

With the implementation of CCPs, the cancer diagnostic process has improved considerably.^36^ However, the present study shows that there is still a substantial proportion of patients where the GP rates the overall diagnostic process to be poor. Efforts should focus on improving this. Both the symptom duration and especially presenting with non-specific symptoms continually present challenges. This finding is despite the implementation of NSSC-CCP in Denmark in 2012, which was meant to improve the diagnosis of cancer in patients with non-specific symptoms.^15^ However, there have been large regional and intraregional variations in implementation of NSSC-CCP.^31^

Better ways of managing patients presenting in general practice with potential non-specific cancer symptoms are necessary. An important tool could be to lower the waiting time for investigations that potentially detect cancer and also when cancer is not explicitly suspected.

Also, artificial intelligence, the development and implementation of new diagnostic tests, and the possibility of referrals based on a GP’s gut feeling alone are initiatives that could improve the diagnostic process for cancer. Moreover, updated knowledge on patients’ healthcare-seeking behaviour and potential barriers to consulting a GP is needed, both to help patients navigate the healthcare system and to develop targeted initiatives addressing both patient healthcare-seeking and the organisation of general practice.

## Data

Access to data is prohibited due to legislation.
